# Supplementary material for: Mechanisms governing the pioneering and redistribution capabilities of the non-classical pioneer PU.1
Source: Nat Commun. 2020 Jan 21;11:402. doi: 10.1038/s41467-019-13960-2 (PMC6972792; doi:10.1038/s41467-019-13960-2)
Supplement: Supplementary file 7 — Source data [file 41467_2019_13960_MOESM7_ESM.zip › Source_Data/FigureS2/FigureS2B_MotifScanOutput/KmeansCluster12/homerResults/motif33.similar.html]

motif33

## Information for motif33

G
A
C
T
T
C
G
A
C
A
T
G
G
A
T
C
C
G
T
A
C
T
A
G
G
A
T
C
G
C
T
A
A
T
C
G
G
T
A
C
  
Reverse Opposite:  

C
A
T
G
T
A
G
C
C
G
A
T
C
T
A
G
G
A
T
C
G
C
A
T
C
T
A
G
G
T
A
C
A
G
C
T
C
T
G
A
  

|  |  |
| --- | --- |
| p-value: | 1e-17 |
| log p-value: | -3.936e+01 |
| Information Content per bp: | 1.650 |
| Number of Target Sequences with motif | 321.0 |
| Percentage of Target Sequences with motif | 17.86% |
| Number of Background Sequences with motif | 5026.0 |
| Percentage of Background Sequences with motif | 10.92% |
| Average Position of motif in Targets | 98.8 +/- 58.5bp |
| Average Position of motif in Background | 102.1 +/- 67.0bp |
| Strand Bias (log2 ratio + to - strand density) | -0.2 |
| Multiplicity (# of sites on avg that occur together) | 1.10 |
| Motif File: | file (matrix) reverse opposite |

### Similar de novo motifs found

|  |  |  |  |  |  |  |  |
| --- | --- | --- | --- | --- | --- | --- | --- |
| Rank | Match Score | Redundant Motif | P-value | log P-value | % of Targets | % of Background | Motif file |
| 1 | 0.754 | G T A C A C G T A C T G G T A C G A C T C T A G G A T C A C G T | 1e-16 | -38.914480 | 29.33% | 20.67% | motif file (matrix) |
| 2 | 0.783 | A C T G A G T C C G T A C T A G G T C A G C T A A C T G | 1e-15 | -35.423398 | 33.67% | 24.97% | motif file (matrix) |
